# Neurological Manifestations in COVID-19: An Unrecognized Crisis in Our Elderly?

**DOI:** 10.20900/agmr20210013

**Published:** 2021-06-16

**Authors:** Karl Krupp, Purnima Madhivanan, William D. “Scott” Killgore, John M. Ruiz, Scott Carvajal, Bruce M. Coull, Michael A. Grandner

**Affiliations:** 1 Mel & Enid Zuckerman College of Public Health, University of Arizona, Tucson, AZ 85724, USA; 2 Public Health Research Institute of India, Mysore, Karnataka 560020, India; 3 College of Medicine, University of Arizona, Tucson, AZ 85724, USA

**Keywords:** elderly, aging, neurological, COVID-19, SARS-CoV-2

## Abstract

As of December 2020, there were more than 900,000 COVID-19 hospitalizations in the US with about 414,000 among individuals aged 65 years and older. Recent evidence suggests a growing number of older patients continue to suffer serious neurological comorbidities including polyneuropathy, cerebrovascular disease, central nervous system infection, cognitive deficits, and fatigue following discharge. Studies suggest that complaints manifest late in disease and persist beyond resolution of acute COVID-19 symptoms. Recent research reports that neurocognitive symptoms are correlated with severe disease, older age, male gender, and comorbidities including hypertension, renal failure, and neoplastic disease. The underlying causes are unclear, but current hypotheses include hypoxic-ischemic brain injury, immunopathological mechanisms, and neurotropism of SARS-CoV-2 infection. There is a pressing need for more research into the underlying mechanisms of post-COVID-19 neurological sequela, particularly in the elderly, a population already burdened with neurocognitive disorders.

As of December 2020, there were almost 80 million confirmed COVID-19 cases worldwide, with the heaviest burden of hospitalizations and deaths among individuals 60 years and older [1,2]. While the main focus has been on the high mortality in this age group, less attention has been paid to mounting reports of persistent post-COVID-19-related neurological symptoms including myalgic encephalomyelitis, seizures, motor and sensory deficits, delirium, psychosis, strokes, and peripheral nerve damage [3]. The scope of COVID-19-related neurocognitive morbidity is poorly understood; studies report rates ranging from 4 to 57% of hospitalized patients [4]. In about a third of cases, neurological symptoms persist beyond hospital discharge and are often accompanied by nonspecific complaints of fatigue, nonrestorative sleep, and attention problems [3,5]. A recent study of Italian COVID-19 patients found they were independently correlated with severe disease, older age, male gender, and non-COVID-19 comorbidities including hypertension, renal failure, and neoplastic disease [6]. Since SARS-CoV-2 is a novel virus, the outlook for these patients is unknown.

A recent study by the Imperial College London analyzing data from 84,285 recovered COVID-19 patients, found “significant cognitive deficits after controlling for age, gender, education level, income, racial-ethnic group, and pre-existing medical disorders” [7]. Patients on mechanical ventilation suffered the worst effects with a 0.57 standard deviation (SD) global composite score reduction equivalent to an average 10-year decline in global performance compared to controls. Standardized differences were larger than the mean deficit for people reporting previous strokes (−0.40 SDs) or learning disabilities (−0.49 SDs). While caution is warranted in interpreting these findings, they are consistent with other research reporting cognitive impairment following COVID-19 disease. Studies from China found that recovered COVID-19 patients exhibited cognitive dysfunction in the sustained attention domain as revealed by Continuous Performance Testing [8]. Data from previous coronavirus epidemics also suggests a potential for long term COVID-19 neurological impairment among vulnerable seniors [9,10].

Hypothesized mechanisms for COVID-19 neurocognitive symptoms include hypoxic-ischemic injury resulting from severe lung damage, immunopathological mechanisms, and neurotropism of SARS-CoV-2 infection [11,12]. Ample evidence from other age-related hypoxic conditions supports a role for oxygen deprivation in COVID-19-related neuropathology [13]. Less data, however, supports other suggested mechanisms. A recent study did note a disease-associated increase in brain microthrombi and cerebral micro-structural alterations in the hippocampus and other brain areas that appeared to be linked to immunopathological causes [14]. Studies using animal models of coronavirus infection have also reported immune-mediated, chronic demyelinating disease similar to multiple sclerosis in humans [15,16]. Other research using mouse-adapted strains of SARS-CoV, found widespread neuronal infection following virus entry via an olfactory route [17]. Post-mortem studies of COVID-19 patients also found demyelinating lesions in the brain and spine, and detected the SARS-CoV-2 virus in the central nervous system (CNS), suggesting the brain and CNS may be extra-pulmonary targets of COVID-19 disease [17–21].

There is a pressing need for longitudinal research among recovered COVID-19 patients to assess the prevalence and persistence of post-COVID-19 neurological symptoms. Studies have yet to explore the relationship between pre-existing neurocognitive disorders and post-COVID-19-related neurological complaints. There is also little data on how long symptoms persist, or if they negatively impact cognitive trajectories in patients with dementia or other neurocognitive disorders. Neurological and cognitive impairments are associated with reduced quality of life, increased caregiver burden, and ultimately, loss of independent living. There is therefore a public health need for a more systematic assessment of cognitive function in patients recovering from COVID-19. The field is currently challenged however, by a lack of precision in case definitions, and poor consensus around the best methods for measuring specific COVID-19 deficits [22].

What do we need in a neurocognitive research agenda for COVID-19? Clearly, the gaps in our understanding of the disease extend well beyond basic pathophysiology and clinical management. We are entering a period where large numbers of elderly COVID-19 patients are being discharged into the community following extended hospitalizations. The US CDC reports that as many as 9% of those patients are readmitted within two months of treatment [23]. Among first-time readmissions, 3.2% are for CNS, mental, behavioral or neurodevelopmental complaints; and that rises to 4.6% in second readmissions [24]. Given the already overwhelming pandemic-related stress on hospital systems, the prospect of a large and growing number of elderly COVID-19 patients having multiple readmissions, should give us all concern. Following the SARS-CoV-1 epidemic of the 2000’s, it was estimated that approximately 3% of survivors required inpatient rehabilitation, and 46% needed some type of outpatient rehabilitative services [25]. Understanding how we can manage this daunting challenge should be a priority question for healthcare systems researchers. Studies assessing the post-pandemic impact of COVID-19 cognitive and neurological disorders on caregiver stress, chronic disease self-management, and the mitigation of accidents and injuries in seniors, is also needed. While we are presently in the middle of one of the most serious healthcare crises in recent memory, the time for planning for post-pandemic challenges is short. Setting an agenda for addressing the needs of seniors suffering the lingering effects of COVID-19 should be a pressing public health priority.
